# Vanishing bone metastasis: pictorial essay

**DOI:** 10.1590/0100-3984.2020.0124

**Published:** 2021

**Authors:** Erina Megumi Nagaya Fukamizu, Adriano Seabra, Deborah Yukiko Otto, Marcio Valente Yamada Sawamura, Marcelo Bordalo-Rodrigues, Paulo Victor Partezani Helito

**Affiliations:** 1 Faculdade de Medicina da Universidade de São Paulo (FMUSP), São Paulo, SP, Brazil.

**Keywords:** Neoplasm metastasis/physiopathology, Bone and bones/diagnostic imaging, Tomography, X-ray computed, Diagnostic errors, Venous thrombosis, Collateral circulation, Metástase neoplásica/fisiopatologia, Osso e ossos/diagnóstico por imagem, Tomografia computadorizada, Erros de diagnóstico, Trombose venosa, Circulação colateral

## Abstract

Vanishing bone metastasis (pseudopathological vertebral body enhancement) is a pitfall in the interpretation of contrast-enhanced computed tomography (CT) scans of patients with thoracic vein obstruction, mainly in the superior vena cava and brachiocephalic veins, typically being related to thrombosis due to malignant tumors. On the basis of the CT findings, pseudopathological vertebral body enhancement can be misdiagnosed as sclerotic bone metastasis, leading to unnecessary treatment. Although not rare, pseudopathological vertebral body enhancement is usually underdiagnosed by radiologists. The aim of this study is to review the pathophysiology of this phenomenon, illustrating the most common collateral venous pathways in thoracic vein obstruction and making the correlation with the CT findings.

## Abstract (pt)

*Vanishing bone metastasis* é um pitfall na tomografia computadorizada (TC) com contraste em pacientes com obstrução das veias torácicas, principalmente das veias cava superior e braquiocefálica, geralmente ocasionada por tumores malignos. Na TC, aparece como um realce pseudopatológico do corpo vertebral e pode ser erroneamente interpretado como metástase óssea esclerótica, levando a tratamento desnecessário. Embora não seja raro, esse achado geralmente é subdiagnosticado pelos radiologistas. O objetivo deste estudo é revisar a fisiopatologia desse fenômeno, ilustrando as vias venosas colaterais mais comuns na obstrução da veia torácica e correlacionar com os achados da TC.

## INTRODUCTION

Vanishing bone metastasis (VBM) is pseudopathological enhancement of bone, particularly of vertebral bodies, on intravenous contrast-enhanced computed tomography (CT) in patients with thoracic vein obstruction, especially of the superior vena cava and brachiocephalic veins. This change is caused by blood congestion in the paravertebral venous plexus, leading to the accumulation of iodinated contrast within the bone, thus simulating a sclerotic lesion. Given the association of malignant mediastinal tumors with thrombotic conditions, this type of alteration may be misdiagnosed as bone metastasis in a cancer patient, resulting in unnecessary biopsies or a false-positive diagnosis of disease progression^(1)^.

Although it is not a common finding, VBM is of great importance for the interpretation of oncology examinations^(1,2)^. Therefore, radiologists should therefore be familiar with the phenomenon. The aim of this article is to review the pathophysiology of VBM by illustrating the most common collateral venous pathways in thoracic vein obstruction and the main imaging findings, with an emphasis on the CT findings.

## PATHOPHYSIOLOGY

The thoracic veins are responsible for the drainage of blood from the head, neck, and upper limbs. When blood flow is impaired by thrombosis or extrinsic compression, there is congestion in the vertebral venous complex and blood is redirected by collateral pathways^(3)^. Although more commonly described in patients with thrombosis, as a result of malignant tumor in 80% of cases^(1)^, a case of VBM was reported in a patient without thrombosis but with extrinsic compression of the brachiocephalic vein between the sternum and the aortic arch^(4)^.

Alternative pathways of venous circulation to superior vena cava obstruction are well reported in the literature, including the lateral thoracic veins, internal thoracic veins, azygos vein, hemiazygos vein, and thoracoabdominal veins^(3)^.

There have been few studies describing the VBM phenomenon. Reflux of contrast medium in the vertebral venous plexus occurs most commonly in the basivertebral vein. The presentation of the phenomenon depends on the site of contrast injection, the infusion rate of contrast medium, and the level of the obstruction^(2,5,6)^. Kim et al.^(5)^ observed that vertebral enhancement appeared only when the intravenous contrast was injected into the arm ipsilateral to the obstructed vein and attributed that to an increase in intravenous pressure, which increased the reflux of contrast in the vertebral body. Beritto et al.^(6)^ agreed with that theory, reporting that a higher rate of intravenous contrast infusion increases the pressure in the collateral venous pathways, thus increasing the enhancement of the vertebral body.

## ANATOMY OF THE VERTEBRAL VENOUS PLEXUS

**Figure 1.**
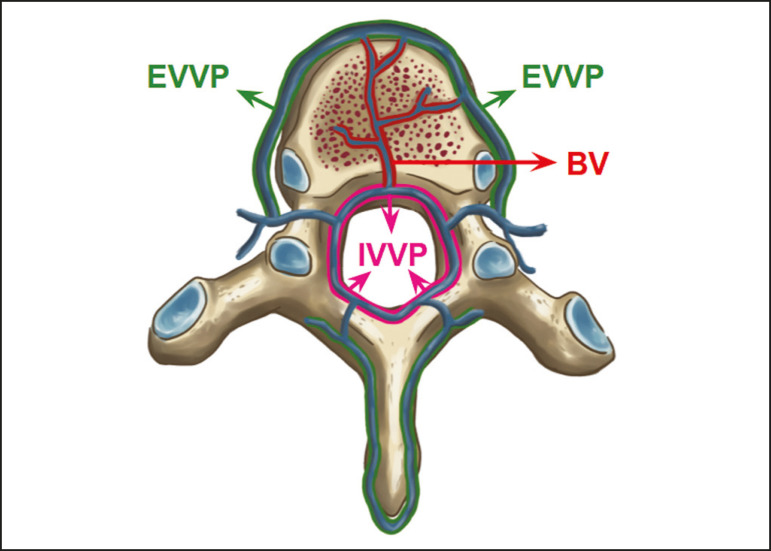
Diagram illustrating the vertebral venous plexus, highlighting the basivertebral vein (BV), external vertebral venous plexus (EVVP), and internal vertebral venous plexus (IVVP).

**Figure 2.**
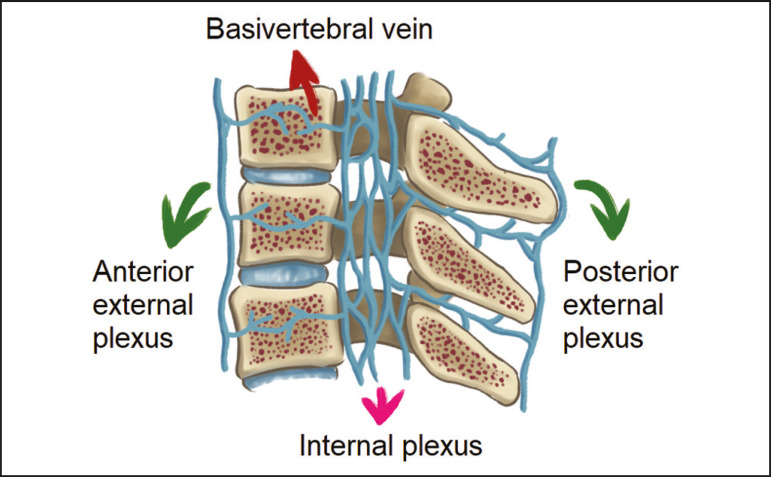
Diagram illustrating the vertebral venous plexus in the sagittal plane.

As reported by Stringer et al.^(7)^, Batson classified the vertebral veins as follows: an internal vertebral venous plexus within the vertebral canal that surrounds the dura mater of the spinal cord; the basivertebral veins within the vertebral bodies; and an external vertebral venous plexus around the spine. Those elements are illustrated in Figures 1 and 2.

The internal vertebral venous plexus and the basivertebral veins do not have valves; so they have reversible flow direction. In some cases, valves have been observed in the external vertebral venous plexus^(7)^.

The external vertebral venous plexus is divided into the anterior plexus and the posterior plexus. The anterior plexus anastomoses with the basivertebral veins, the intervertebral veins, and tributary veins of the vertebral bodies. The posterior plexus anastomoses with the internal vertebral venous plexus and terminates in the vertebral, lumbar, and intercostal veins^(2)^.

The internal vertebral venous plexus, known as Batson’s plexus, is responsible for venous drainage of the brain and spinal cord, being divided into two anterior and two posterior longitudinal veins^(7)^. The paravertebral venous plexus forms multiple anastomoses via segmental veins, communicating with the left brachiocephalic vein, azygos-hemiazygos vein, and left renal vein or inferior vena cava^(7)^.

**Figure 3.**
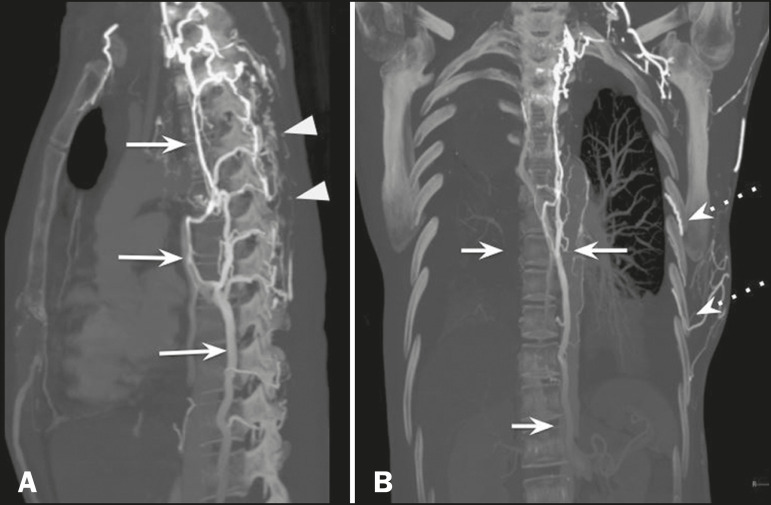
Superior vena cava obstruction, with consequent prominence of the azygos-hemiazygos system (arrows). Observe the external paravertebral venous plexuses (arrowheads) and the intercostal collateral veins (dotted arrows).

**Figure 4.**
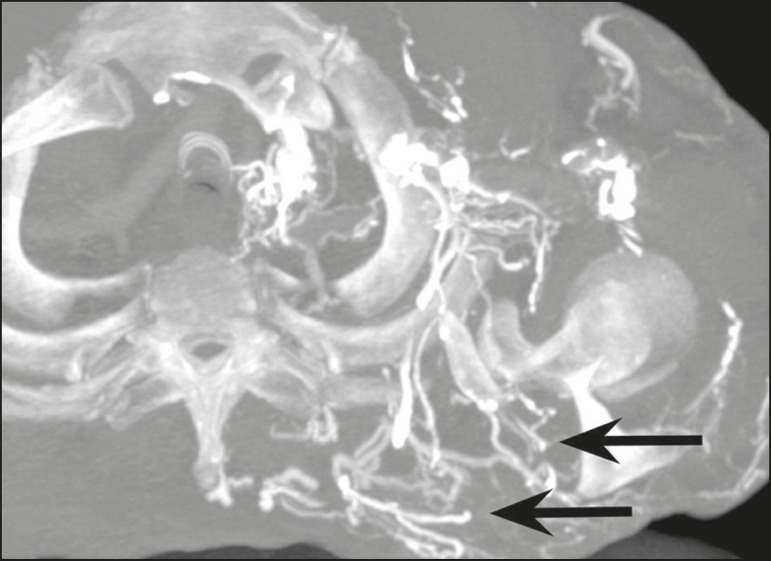
Patient with superior vena cava thrombosis and subscapular collateral vascular system ectasia to the left (arrows).

**Figure 5.**
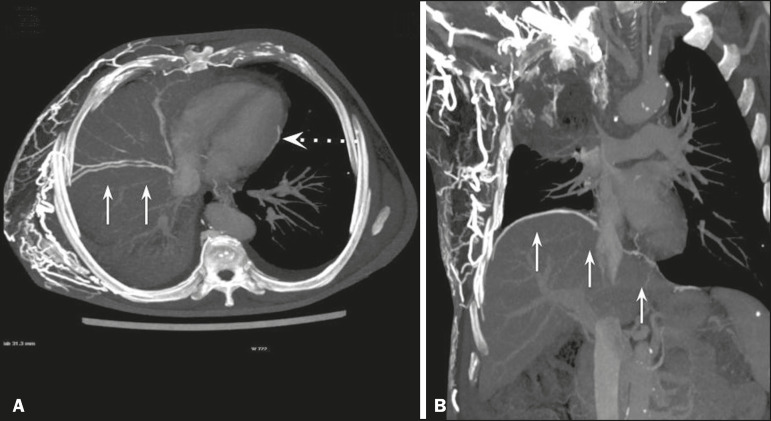
The mediastinal, pericardial, and pericardiophrenic collateral veins drain into the inferior vena cava via the inferior phrenic and transhepatic collateral veins. Arrows: pericardiophrenic collateral veins. Dotted arrow: pericardial collateral veins.

**Figure 6.**
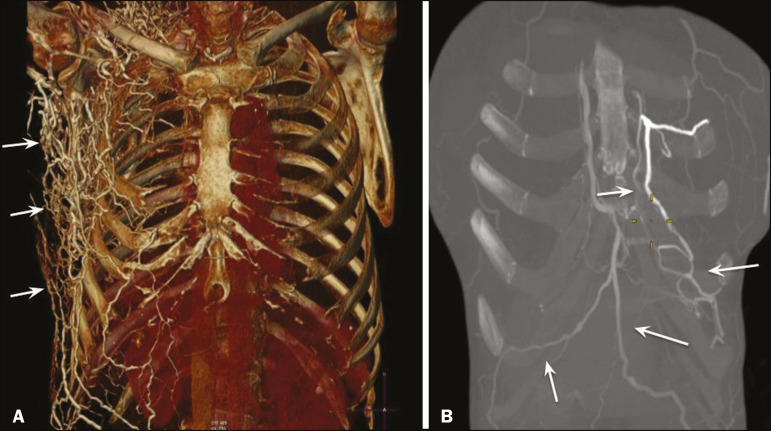
Superficial thoracoabdominal plexus collateral venous pathway (arrows).

**Figure 7.**
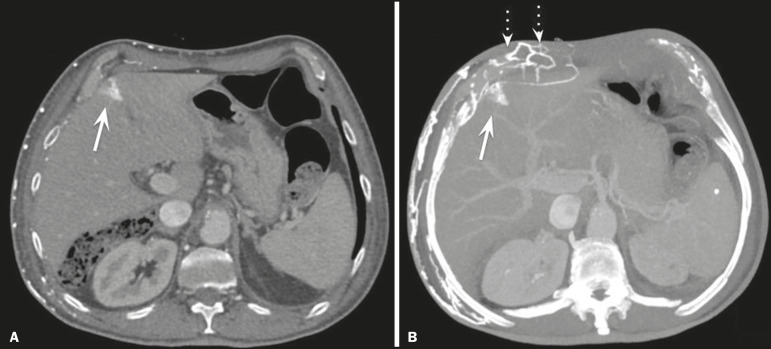
The collateral thoracic veins drain into the left hepatic portal vein to reach the inferior vena cava, resulting in an area of increased contrast in the left hepatic lobe. Arrows: hepatic “hot spot” sign. Dotted arrows: superficial thoracic collateral veins.

In the scenario of occlusion or subocclusion of the superior vena cava, brachiocephalic vein, subclavian vein, azygos vein, or hemiazygos vein, the collateral veins find a way to drain into the inferior vena cava^(3)^. The four main collateral venous pathways are the azygos-hemiazygos system (Figure 3); the vertebral and subscapular plexuses (Figure 4); the mediastinal, esophageal, and diaphragmatic plexuses (Figure 5); and the internal thoracic and superficial thoracoabdominal plexuses (Figures 6 and 7). However, it is quite difficult to predict the venous drainage pattern because of individual variations in venous anatomy^(7)^.

## IMAGING FINDINGS

**Figure 8.**
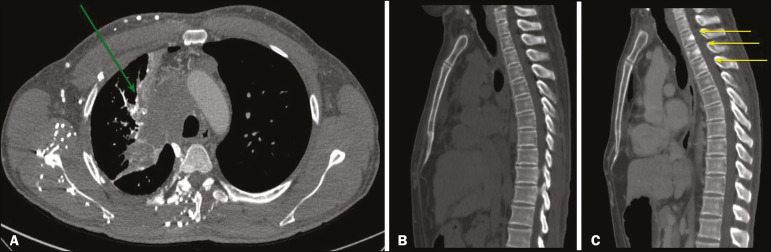
**A:** Chest CT with intravenous contrast in a 50-year-old male patient diagnosed with small cell carcinoma evolving to superior vena cava thrombosis (arrow). **B,C:** Unenhanced and contrast-enhanced CT images, respectively. The unenhanced image shows preserved vertebral bodies, and the contrast-enhanced image shows enhancement of the upper thoracic vertebral bodies, due to reflux of contrast medium by the vertebral venous plexus (arrows).

**Figure 9.**
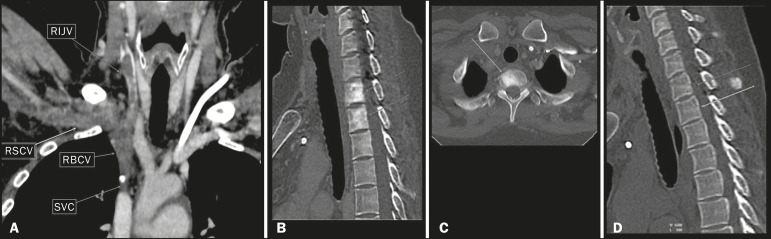
A 34-year-old female patient who was receiving peripheral parenteral nutrition after massive enterectomy, because of mesenteric ischemia three years prior, and evolved to mild pain in the cervical and right supraclavicular regions. **A:** CT angiography showing thrombosis of the superior vena cava (SVC). **B,C:** In the images that include the thoracic spine, hyperdense areas can be seen in the vertebral bodies of the upper thoracic segment. **D:** On an unenhanced follow-up CT angiography image, no such hyperdense areas were seen. RIJV, right internal jugular vein; RSCV, right subclavian vein; RBCV, right brachiocephalic vein.

When there is suspicion of VBM, the evaluation begins with the identification of bone alterations in a contrast-enhanced imaging examination, followed by the characterization of bone pseudolesions and the identification of a condition-typically thrombosis-that alters the venous flow (Figures 8 and 9). Bone pseudolesions occur preferentially in vertebral bodies. Although they may affect pedicles, laminae, and spinous processes, the involvement of those sites is rarely isolated. The asymmetric or unilateral contrast enhancement pattern of a vertebra has been associated with the side of contrast administration^(2)^. Within the vertebral body, the alteration commonly affects the middle third and central portions, corresponding to the anatomical location of the basivertebral vein. Although there is a preference for vertebrae, such alterations may also be observed in other bones, such as the sternum^(5)^.

The hyperattenuation of pseudolesions may have a well-defined or poorly defined aspect, presenting larger dimensions and lower density in relation to how long acquisition is delayed. The shape of a pseudolesion ranges from oval and focal to polygonal and more diffuse^(5)^ and may involve the vertebral body diffusely. The identification of ectatic vessels within the vertebra or in the soft tissues adjacent to the pseudolesion increases diagnostic confidence^(2)^.

As previously mentioned, identification of thrombosis or alterations that predispose to collateral circulation is fundamental for the diagnosis of VBM. On intravenous contrast-enhanced CT scans, thrombosis is easily diagnosed by the presence of central or eccentric filling defects in the veins. In cases of recent thrombosis, the size of the vessel may be increased, whereas in more chronic cases, there is usually a diffuse reduction in caliber. It should also be highlighted that thrombi resulting from malignant mediastinal tumors appear with lobulated contours and may have heterogeneous contrast enhancement^(8)^. Identifying and delineating the location of obstruction is also relevant for the interpretation of bone lesions. In general, when there is obstruction in the superior vena cava or near the confluence of the superior vena cava and the innominate vein, the phenomenon is observed in the lower cervical spine or upper thoracic spine. However, when there is involvement of the hemiazygos veins, the pseudolesion is more evident in the lower thoracic spine^(2,9)^. If diagnostic uncertainty persists, making the correlation with unenhanced CT (a recent examination or a new acquisition) is sufficient to define the vascular nature of the pseudolesion and exclude a true bone lesion^(4,5)^.

## CONCLUSION

The VBM phenomenon is still an underdiagnosed condition. In most cases, there is occlusion of the superior vena cava or brachiocephalic vein and the typical radiological finding is a focus or hyperattenuating area in the thoracic vertebral body on contrast-enhanced CT, representing enhancement of the venous plexus collateral system in the vertebral body. The condition can be diagnosed on the basis of imaging findings, both by bone alteration characteristics and by vascular findings. In cases of uncertainty, making the correlation with unenhanced CT is sufficient to define the diagnosis.
